# Lack of efficacy of standard doses of ivermectin in severe COVID-19 patients

**DOI:** 10.1371/journal.pone.0242184

**Published:** 2020-11-11

**Authors:** Daniel Camprubí, Alex Almuedo-Riera, Helena Martí-Soler, Alex Soriano, Juan Carlos Hurtado, Carme Subirà, Berta Grau-Pujol, Alejandro Krolewiecki, Jose Muñoz

**Affiliations:** 1 Barcelona Institute for Global Health (ISGlobal), Hospital Clínic—Universitat de Barcelona, Barcelona, Spain; 2 Infectious Diseases Department, Hospital Clínic—Universitat de Barcelona, Barcelona, Spain; 3 Microbiology Department, Hospital Clínic—Universitat de Barcelona, Barcelona, Spain; 4 Instituto de Investigaciones de Enfermedades Tropicales, Universidad Nacional de Salta/CONICET, Orán, Argentina; Netherlands Cancer Institute, NETHERLANDS

## Abstract

Ivermectin has recently shown efficacy against SARS-CoV-2 in-vitro. We retrospectively reviewed severe COVID-19 patients receiving standard doses of ivermectin and we compared clinical and microbiological outcomes with a similar group of patients not receiving ivermectin. No differences were found between groups. We recommend the evaluation of high-doses of ivermectin in randomized trials against SARS-CoV-2.

## Introduction

Several months after the beginning of the spread of severe acute respiratory syndrome coronavirus 2 (SARS-CoV-2), few therapeutic agents have proven their efficacy in human clinical trials [1, 2]. Several repurposed drugs with antiviral effect have been tested outside the scope of the initial approved medical use, such as lopinavir/ritonavir, hydroxychloroquine or azithromycin [3]. Researchers in Australia have shown ivermectin (IVM) to be active against SARS-CoV-2 in cell cultures by drastically reducing viral RNA at 48h [4]. The concentrations tested in these in-vitro assays are equivalent to more than 50-fold the normal C_max_ achieved with a standard single dose of IVM 200 μg/kg, raising concerns about the effective dose of IVM for treating SARS-CoV-2 infection in humans and its tolerability [5]. The hypothesis of our study was that standard doses of IVM to treat strongyloidiasis (200ug/kg single dose) were not efficacious to treat patients with SARS-CoV-2 pneumonia. We evaluated clinical and microbiological outcomes of 13 patients with confirmed SARS-CoV-2 severe infection receiving standard doses of f IVM in comparison with a similar group of patients not receiving IVM.

## Methods

For this retrospective study, we identified hospitalized patients diagnosed with SARS-CoV-2 infection receiving IVM between March 10^th^ and 30^th^ 2020 in Hospital Clinic in Barcelona, Spain. Patients from countries endemic for *Strongyloides stercoralis* receiving immunosuppressant drugs such as corticosteroids or tocilizumab for COVID-19 were empirically treated with IVM 200μg/kg, single dose, following standard hospital procedures based on international recommendations (IVM group) [6]. Once identified, an equal number of COVID-19 patients with similar baseline characteristics and immunosuppressive treatment but not receiving IVM (non-IVM group) were selected as a comparator group.

Patients diagnosed with SARS-CoV-2 infection were admitted and quarantined in the ward during the study period. Diagnosis of COVID-19 was performed with IgM and IgG antibodies rapid diagnostic test (VivaDiag™ COVID-19 IgM/IgG Rapid Test) and/or polymerase chain reaction (PCR) assay in nasopharyngeal swab samples. A full biochemistry and haematology profile including C-reactive protein, D-dimer and ferritin and a chest X-ray was performed to all patients at hospitalization. Nasopharyngeal swab was repeated for standard control 6–12 days after the beginning of the antiviral treatment.

Data were obtained as part of standard care, to create a fully anonymized database. Categorical variables were expressed as absolute frequency and percentage and compared with chi-square test or Fisher’s exact test. Continuous variables were expressed as median and interquartile range (IQR) and compared with Mann-Whitney-Wilcoxon test. The statistical analysis was carried out using Stata 15 (StataCorp.2017).

This study was approved by the Ethics Committee of Hospital Clinic of Barcelona (HCB/2020/0475), who waived the requirement for informed consent, due to the retrospective nature of the study.

## Results

During the study period a total of 13 severe COVID-19 patients receiving immunosuppressant therapy were treated with IVM at 200 μg/kg, single dose. In the IVM group, 5 (38.5%) patients were treated with tocilizumab, 3 (23.1%) with high doses of steroids, 3 (23.1%) with both tocilizumab and steroids, and 2 (15.3%) with tocilizumab, steroids and anakinra. Five patients required admission to an ICU. IVM was administered a median of 12 (IQR 8–18) days after the initiation of symptoms. In the non-IVM group, six (46.2%) patients were treated with tocilizumab and steroids, 2 (15.3%) with anakinra and steroids, 2 (15.3%) with tocilizumab, 2 (15.3%) with high doses of steroids and 1 with siltuximab.

Following hospital protocols at that moment, all patients received hydroxychloroquine and azithromycin. All patients in the control group and 12 up to the 13 patients in the IVM group were also treated with lopinavir/ritonavir. One patient in the IVM group did not receive lopinavir/ritonavir due to diarrhea. Two patients in the IVM group and one in the control group were also treated with remdesivir and one patient in the IVM group and two in the control group received beta-interferon. Comparison of baseline characteristics, clinical presentation, treatment and outcomes between COVID-19 patients treated with and without IVM is shown in Table 1. Although no significant differences in baseline characteristics were observed between groups, a higher proportion of patients in the IVM group required admission to an intensive care unit (ICU) (69% vs 38% in the non-IVM group) (Table 1).

**Table 1:** Comparison of baseline characteristics, clinical presentation, treatment and outcomes of COVID-19 patients treated with and without ivermectin (IVM).

	No IVM (n = 13)	IVM (n = 13)	p-value
**Baseline characteristics**			
Sex (female)	5 (38.5)	4 (30.8)	1.000 ^μ^
Age	54 [48–58]	43 [41–49]	0.117
Origin			0.006 ^μ^
• Europe	7 (53.8)	0 (0)	
• South-America	5 (38.5)	10 (76.9)	
• Asia	1 (7.7)	3 (23.1)	
Comorbidities	12 (92.3)	9 (69.2)	0.320
**Clinical presentation**			
Cough	10 (76.9)	11 (84.6)	1.000
Dyspnea	11 (84.6)	8 (61.5)	0.376
Fever	13 (100)	13 (100)	--
Abdominal symptoms	6 (46.1)	4 (30.8)	0.687
Days before admission^1^	7 [6–9]	7 [5–9]	0.816
Radiological pattern			0.185 ^μ^
• Interstitial pattern	2 (15.4)	5 (38.5)	
• Patchy infiltrates	5 (38.5)	6 (46.1)	
• Mixed pattern	6 (46.1)	2 (15.4)	
CRP	12.05 [6.48-21-72]	14.22 [8.37–20.68]	0.644
LDH	426 [333–501]	383 [301–418]	0.317
D-dimer	500 [400–800]	500 [400–1000]	0.836
Ferritin	1243 [654.25–2259.75]	1101 [477.5–1434.5]	0.751
Lymphocytes	800 [500–900]	900 [500–1200]	0.279
NLR	11.2 [6.9–13.4]	4.9 [2.5–10.0]	0.106
Eosinphils^ß^	0	0 (0–200)	--
**Pharmacological treatment**			
Antiviral agents	13 (100)	12 (92.3)	1.000
IS treatment	13 (100)	13 (100)	--
**Steroids**	10 (76.9)	8 (61.5)	0.671
Days until steroids treatment	9 [8–13]	8.5 [6.75–10.75]	0.531
**Anti-IL treatment**	11 (84.6)	10 (76.9)	1.000
• Anti IL-6 (tocilizumab, siltuximab)	9 (69.2)	10 (76.9)	1.000
• Anti IL-1 (anakinra)	2 (15.4)	2 (15.4)	1.000 ^μ^
Days until anti-IL treatment	9 [7.5–13.5]	9.5 [7.5–13.5]	0.671
**Supportive treatment**			
Maximum FiO2	60 [23–60]	40 [23–60]	0.529
NIV/HFNC	4 (30.8)	2 (15.4)	0.645 ^μ^
ETI + MV	5 (38.5)	3 (23.1)	0.671 ^μ^
Admission to ICU	9 (69.2)	5 (38.5)	0.238
**Outcomes**			
Other severe adverse events^4^	4 (30.8)	3 (23.1)	1.000 ^μ^
**Positive PCR 3–5 days after IVM**^**2**^	4 (30.8)	5 (38.5)	1.000 ^μ^
Days to naso-pharyngeal swab^3^	19 [15–21]	15 [12–21]	0.382
CRP^3^	0.4 [0.4–2.57]	0.4 [0.5–2.2]	0.368
LDH^3^	300 [277–374]	266 [246.7–327.2]	0.097
D-dimer^3^	1600 [1300–4300]	850 [600–4275]	0.351
Ferritin^3^	1263 [771–1785.5]	816 [414–1031]	0.172
Lymphocytes^3^	1100 [700–1300]	1400 [875–1800]	0.369
NLR^3^	8.38 [3.55–13.75]	3.22 [1.92–9.35]	0.201
Eosinphils^3^^ß^	100 [0–100]	100 [0–125]	0.839
Improvement 8 days after IVM^3^	10 (76.9)	9 (69.2)	1.000 ^μ^
Localization 8 days after IVM^3^			1.000 ^μ^
• Discharged	6 (46.1)	7 (53.8)	
• Hospitalized	4 (30.8)	4 (30.8)	
• ICU	3 (23.1)	2 (15.4)	

N (%) or median [p25-p75].

^(ß)^median (range).

^(μ)^Fisher’s exact test.

^(1)^ Days between symptoms initiation and admission to hospital.

^(2)^ Naso-pharyngeal swab performed between 3 and 5 days after ivermectin treatment (or equivalent time in the non-IVM group).

^(3)^ 8–11 days after IVM treatment (or equivalent time in the non-IVM group).

^(4)^ Other adverse events in patients not receiving IVM: organizing pneumonia (1), acute kidney injury requiring hemodialysis (1), pancreatitis (1) and catheter bacteremia (1). Other adverse events in patients receiving IVM: organizing pneumonia (1), pulmonary embolism (1) and *Strongyloides* infection (1).

CRP: C-reactive protein. ETI+MV: endotracheal intubation + mechanical ventilation. FiO2: Fraction of inspired oxygen. ICU: intensive care unit. IS: immunosuppressant treatment. IL: interleukin. IVM: ivermectin. LDH: lactate dehydrogenase. NIV/HFNC: Non-invasive ventilation / high flow nasal cannula. NLR: Neutrophil-to-lymphocite ratio. PCR: polymerase chain reaction.

No relevant differences in microbiological or clinical outcomes were observed between groups. SARS-CoV-2 PCR from nasopharyngeal swabs performed between 3 and 5 days after receiving ivermectin resulted positive in 5 out of 13 patients in the IVM group (38.5%), and 4/13 in the non-IVM group (30.8%, *p-value* >0.999). A remarkable clinical improvement was observed in 9 (69.2%) participants receiving IVM and in 10 (76.9%) of the non-IVM group, with no differences between groups (*p-value* >0.999), eight to eleven days after IVM treatment (or equivalent time in the non-IVM group).

## Discussion

In our retrospective study, a single dose of 200 μg/kg of IVM did not improve clinical and microbiological outcomes of patients with severe COVID-19, compared to a similar group of patients not receiving IVM. Although IVM may lack of in-vivo effect against SARS-CoV2, in our study the drug was given at late stages of the infection (median 12 days after the beginning of symptoms) and, most importantly, all patients received a standard (200 μg/kg) single dose of the drug, which could be below the IC50 values [4, 5] for SARS-CoV-2 infection.

In the last years, high doses of IVM have been evaluated for the treatment of soil-transmitted helminths [7–10] and as a new vector control tool to reduce malaria transmission in malaria endemic areas [11]. Recent studies have evaluated doses up to 800 μg/kg, given in single dose or three consecutive days [9, 11, 12], showing a good safety profile both in adult and paediatric populations. Subjective ocular problems such as transitory blurred vision appeared, but no severe adverse events were reported with these high doses [11, 12].

These findings, including a recent meta-analysis of the safety of high doses of ivermectin [7], add evidence of the safety of IVM at doses up to 800 μg/kg, which has a safety profile comparable to lower doses of 200 or 400 μg/kg. Moreover, the results of the meta-analysis do not suggest an increased number of adverse events with increasing doses of IVM. The maximum doses of IVM given to study participants have been published in a study with a limited number of participants, in which doses up to 2000 μg/kg were received by 12 participants, showing a similar rate of adverse events than those receiving placebo [13]. However, the antiviral efficacy of these high doses of IVM should be still evaluated in clinical studies, since some authors have recently suggested that in vitro inhibitory concentrations of 5umol/L (those needed for a total eradication of SARS-CoV-2 in in vitro studies) would not be attainable even using high doses of ivermectin (2000ug/kg) [4, 14].

The study has some limitations. Given the retrospective design of the study, possible confounding factors could bias the results of the study, which was addressed by a careful selection of a matched control group. Potential differences between groups might not be detected due to the small sample size and the lack of a quantitative evaluation of the viral response. Activity of some antiviral treatments received cannot be excluded. However, no differences between both study groups should be expected given that antivirial regimens between groups were similar. Another limitation of the study was that disease status at baseline could not be confirmed by PCR in all patients. Nevertheless, all patients presented with symptoms, signs, blood test alterations and radiological findings compatible with COVID-19. Median of time between symptoms onset and hospital admission for patients who were not diagnosed by PCR was 6 days (range 2–9), which was not different to the median of time of those patients diagnosed by PCR.

Finally, in light of the presented results, it is unlikely that the widespread use of IVM at standard doses may have an impact in decreasing the mobility related with COVID-19. We suggest the evaluation of high-doses of IVM in randomized clinical trials to test the efficacy of IVM in COVID-19 patients, especially in early stages of the disease.
