# Correction: Lack of efficacy of standard doses of ivermectin in severe COVID-19 patients

**DOI:** 10.1371/journal.pone.0268667

**Published:** 2022-05-12

**Authors:** Daniel Camprubí, Alex Almuedo-Riera, Helena Martí-Soler, Alex Soriano, Juan Carlos Hurtado, Carme Subirà, Berta Grau-Pujol, Alejandro Krolewiecki, Jose Muñoz

In the Results section, there is an error in the sixth sentence of the second paragraph. The correct sentence is: Although no statistically significant differences in baseline characteristics were observed between groups, 38% of patients in the IVM group and 69% in the non-IVM group were initially admitted to an intensive care unit (ICU) (p = 0.238) (Table 1).

It is noted that a non-significant result does not confirm the absence of an effect. Therefore, the authors provide the following corrections to errors in the title, and in the Discussion section, in the first sentence of the first paragraph.

The correct title of the manuscript is: Lack of evidence for efficacy of standard doses of ivermectin in severe COVID-19 patients.

The correct citation is: Camprubí D, Almuedo-Riera A, Martí-Soler H, Soriano A, Hurtado JC, Subirà C, et al. (2020) Lack of evidence for efficacy of standard doses of ivermectin in severe COVID-19 patients. PLoS ONE 15(11): e0242184. https://doi.org/10.1371/journal.pone.0242184

In the Discussion section, in the first sentence of the first paragraph, the correct sentence is: In our retrospective study, we did not observe significant differences in clinical and microbiological outcomes of patients with severe COVID-19 receiving a single dose of 200 μg/kg of IVM compared to a similar group of patients not receiving the drug.
